# Complete mitochondrial genome of *Scythris sinensis* (Lepidoptera: Scythrididae)

**DOI:** 10.1080/23802359.2020.1780990

**Published:** 2020-06-17

**Authors:** Jeong Sun Park, Min Jee Kim, Sung-Soo Kim, Iksoo Kim

**Affiliations:** a Department of Applied Biology, College of Agricultural and Life Sciences, Chonnam National University, Gwangju, Republic of Korea; b Herbal Medicine Resources Research Center, Korea Institute of Oriental Medicine, Naju, Republic of Korea; c Research Institute for East Asian Environment and Biology, Seoul, Republic of Korea

**Keywords:** Mitochondrial genome, Scythrididae, *Scythris sinensis*, phylogeny

## Abstract

The complete mitochondrial genome (mitogenome) of *Scythris sinensis* Felder & Rogenhofer, 1775 (Lepidoptera: Scythrididae) was 15,216 bp with a typical set of genes (13 protein-coding genes [PCGs], 2 rRNA genes, and 22 tRNA genes) and one non-coding region, with an arrangement identical to that observed in most lepidopteran genomes. Twelve PCGs had the typical ATN start codon, whereas *COI* had the atypical CGA codon that is frequently found in the start region of the lepidopteran *COI*. The 271-bp long A + T-rich region was the shortest among sequenced Gelechioidea, which ranged from 290 – 375 bp. Phylogenetic analyses with concatenated sequences of the 13 PCGs, two RNA genes, and 22 tRNA genes using the Bayesian inference (BI) method, placed *S. sinensis* in the Scythrididae, as a sister to the family Stathmopodidae. The nodal support for this sister relationship was the highest at Bayesian posterior probabilities = 1.

*Scythris sinensis* Felder & Rogenhofer, 1775 (Lepidoptera: Scythrididae) belongs to Scythrididae. The species is found in eastern Asia including Korea, Europe, central Russia, southern Siberia, and eastern North America (Bengtsson 1997; Nupponen et al. 2000; Nupponen and Nupponen 2001; Landry et al. 2013; Bidzilya et al. 2017). The species occurs from May to July in Korea, has a pair of yellow patches on the front wings, and has bright yellow abdominal segments on its abdomen, which is broader in the female (Landry et al. 2013). The larvae of the species feed on *Chenopodium album* and *Atriplex patula* belonging to Chenopodiaceae and live in a loose spinning inside the young leaves at the top of the plant, concealed between the buds and leaves (Malkiewicz and Dobrzański 2011).

An adult male *S. sinensis* was collected from Gyeongju-city, Gyeongsangnam-do Province (35°50′42.0″N, 129°30′13.5″E), South Korea in 2012. This voucher specimen was deposited at the Chonnam National University, Gwangju, Korea, under the accession no. CNU6197. Using DNA extracted from the hind legs, three long overlapping fragments (LFs; *COI*-*ND4*, *ND5*-*lrRNA*, and *lrRNA*-*COI*) were amplified using three sets of primers that were previously published (Kim et al. 2012). Subsequently, these LFs were used as templates to amplify 26 short fragments (Kim et al. 2012).

Phylogenetic analysis was performed using the concatenated nucleotide sequences of 13 protein-coding genes (PCGs), two RNA genes, and 22 tRNA genes of 14 mitogenome sequences from Gelechioidea in Lepidoptera, including that of *S. sinensis*. The Bayesian inference (BI) method implemented in CIPRES Portal v. 3.1 (Miller et al. 2010) was used for phylogenetic analysis.

The complete 15,216-bp mitogenome of *S. sinensis* was composed of typical sets of genes (two rRNAs, 22 tRNAs, and 13 PCGs) and a major non-coding 271 bp A + T-rich region (GenBank accession no. MH230111). The gene arrangement of *S. sinensis* is identical to that of the ditrysian Lepidoptera that have the order *trnM*-*trnI*-*trnQ* (where underlining indicates a gene inversion) between the A + T-rich region and *ND2* (Kim et al. 2010) instead of the ancestral *trnI*-*trnQ*-*trnM* order found in the majority of insects (Boore 1999). The A/T content of the whole mitogenome was 80.9%; however, it varied among the genes as follows: the A + T-rich region, 96.3%; *srRNA*, 85.1%; *lrRNA*, 84.2%; and PCGs, 79.5%. Twelve PCGs had the typical ATN start codon, whereas *COI* had the atypical CGA codon. Ten of the 13 PCGs had a complete stop codon (TAA); however, *COI*, *COII*, and *ND5* had an incomplete stop codon, T.

Phylogenetic analysis resulted in a sister relationship between Scythrididae, represented by the current *S. sinensis* and the Stathmopodidae, represented by *Stathmopoda auriferella*, *Hieromantis kurokoi*, and *Atrijuglans hetaohei,* with the highest nodal support (Bayesian posterior probabilities [BPP] = 1; Figure 1). This sister relationship also was supported by 19 nuclear gene-based analyses (Sohn et al. 2016). Among the three families in Gelechioidea represented by more than one species, Gelechiidae and Stathmopodidae formed strong monophyletic groups (BPP = 1), whereas Oecophoridae, represented by two species, formed a non-monophyletic group (Figure 1). Currently, complete mitogenome sequences are only available from 14 species in Gelechioidea, including that of *S. sinensis*. Thus, more mitogenome sequences from such a diverse taxonomic group are required for further comprehensive phylogenetic inference.

**Figure 1.**
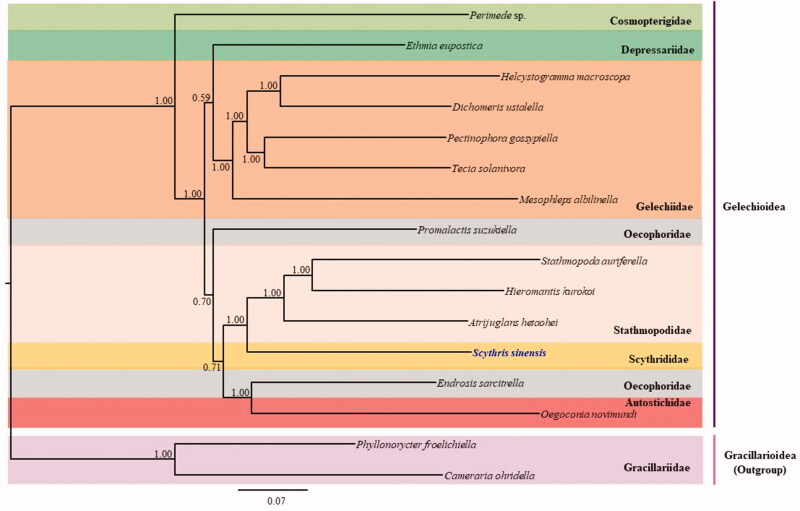
Phylogenetic tree for Gelechioidea. The tree was constructed using nucleotide sequences of 13 protein-coding genes, two rRNA genes, and 22 tRNA genes via the Bayesian inference method. The numbers at each node indicate the Bayesian posterior probabilities. The scale bar indicates the number of substitutions per site. Two species belonging to the family Gracillariidae in Gracillarioidea were used as outgroups. GenBank accession numbers are as follows: *Perimede* sp., KJ508041 (Timmermans et al. 2014); *Ethmia eupostica,* KJ508047 (Timmermans et al. 2014); *Helcystogramma macroscopa*, KT354968 (Ma et al. 2016); *Dichomeris ustalella*, KU366706 (Park et al. 2016b); *Pectinophora gossypiell*, KM225795 (Zhao et al. 2016); *Tecia solanivora*, KT326187 (Ramírez-Ríos et al. 2016); *Mesophleps albilinella*, KU366707 (Park et al. 2016b); *Promalactis suzukiella*, KM875542 (Park et al. 2016c); *Stathmopoda auriferella*, KX138529 (Jeong et al. 2016); *Hieromantis kurokoi*, KU605775 (Park et al. 2016a); *Atrijuglans hetaohei*, KT581634 (Wang et al. 2016); *Endrosis sarcitrella*, KJ508037 (Timmermans et al. 2014); *Oegoconia novimundi*, KJ508036 (Timmermans et al. 2014); *Phyllonorycter froelichiella*, KJ508048 (Timmermans et al. 2014); and *Cameraria ohridella*, KJ508042 (Timmermans et al. 2014).

## Data Availability

The data that support the findings of this study are openly available in Mendeley Data at http://dx.doi.org/10.17632/rdwz62m66w.1
